# Supplementary material for: Fast and Sensitive Analysis of Short- and Long-Chain Perfluoroalkyl Substances in Foods of Animal Origin
Source: Molecules. 2022 Nov 15;27(22):7899. doi: 10.3390/molecules27227899 (PMC9697276; doi:10.3390/molecules27227899)
Supplement: Supplementary file 1 [file molecules-27-07899-s001.zip › molecules-1945374-supplementary.pdf]

Supplementary materials

Table S1. Detailed information about the 63 analysed food samples

**Matrix (n)**	**Species/Category**	**Sample ID**
Liver (34)	Trout	L1
	Trout	L2
	Hen	L3
	Calf	L4
	Calf	L5
	Calf	L6
	Calf	L7
	Calf	L8
	Calf	L9
	Calf	L10
	Calf	L11
	Calf	L12
	Calf	L13
	Calf	L14
	Bullock	L15
	Bullock	L16
	Bullock	L17
	Bullock	L18
	Bullock	L19
	Bullock	L20
	Bullock	L21
	Bullock	L22
	Bullock	L23
	Bullock	L24
	Bovine Adult	L25
	Bovine Adult	L26
	Bovine Adult	L27
	Bovine Adult	L28
	Bovine Adult	L29
	Bovine Adult	L30
	Bovine Adult	L31
	Bovine Adult	L32
	Swine	L33
	Swine	L34
Eggs (6)	Hens	E1
	Hens	E2
	Hens	E3
	Hens	E4
	Hens	E5
	Hens	E6
Muscle (17)	Trout	M1
	Sea Bream	M2
	Salmon	M3
	Trout	M4

	Trout	M5
	Pangasius	M6
	Chicken	M7
	Chicken	M8
	Chicken	M9
	Chicken	M10
	Chicken	M11
	Chicken	M12
	Bovine	M13
	Bovine	M14
	Bovine	M15
	Bovine	M16
	Swine	M17
Milk (6)	Cow	Mi1
	Cow	Mi2
	Cow	Mi3
	Cow	Mi4
	Cow	Mi5
	Cow	Mi6

Table S2. Instrumental mass spectrometry settings for target compounds

Compound	Precursor ion (m/z)	Product ion 1 (m/z)	Collision energy (eV)	Product ion 2 (m/z)	Collision energy (eV)	Retention time (minute)	Internal standard
PFBA	213.0	169.1	-14			5.1	MPFBA
PFPeA	217.0	172	-14			8.8	M5PFPeA
PFHxA	313.0	269.0	-13	119.1	-30	10.0	M5PFHxA
PFHpA	363.0	319.4	-15	169.2	-20	10.8	M4PFHpA
PFOA	413.3	369.0	-15	169.0	-20	11.3	M8PFOA
PFNA	463.0	418.9	-13	168.9	-26	11.8	M9PFNA
PFDA	513.3	469.2	-15	218.9	-22	12.2	M6PFDA
PFUnA	563.0	519.0	-16	319.0	-28	12.5	M7PFUdA
PFDoA	613.0	569.0	-20	318.9	-28	12.8	M2PFDoA
PFBS	299.0	80	-70	98.8	-40	9.5	M3PFBS
PFHxS	399.1	79.9	-85	98.9	-80	11.0	M3PFHxS
PFOS	499.0	80.0	-106	99.0	-80	11.9	M8PFOS
GenX	328.5	284.9	-8	168.9	-17	10.3	M5PFHxA
C6O4	338.9	179.0	-6	112.9	-13	10.6	M5PFHxA
MPFBA	217.0	172.0	-14			5.1	
M5PFPeA	268.0	223.0	-12			8.8	
M5PFHxA	318.0	273.0	-13			10.0	
M4PFHpA	367.0	322.0	-15			10.8	
M8PFOA	421.0	376.0	-14			11.3	
M9PFNA	472.0	427.0	-15			11.8	
M6PFDA	519.0	474.0	-15			12.2	

M7PFUdA	570.0	525.0	-16	12.5
M2PFDoA	614.9	569.9	-16	12.8
M3PFBS	302.0	322.0	-15	9.5
M3PFHxS	402.0	99.0	-70	11.0
M8PFOS	507.0	99.0	-80	11.9

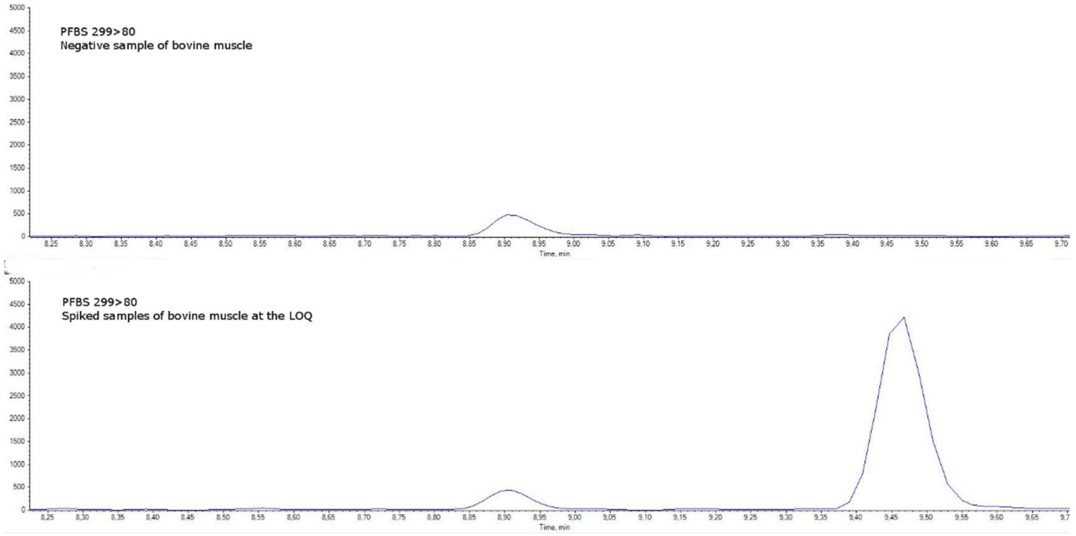

Figure S1. (PFBS quantitative transition 299>80). Comparison between a negative sample of bovine muscle (upper chromatogram) and spiked sample of bovine muscle at the Limit of Quantification (lower chromatogram).

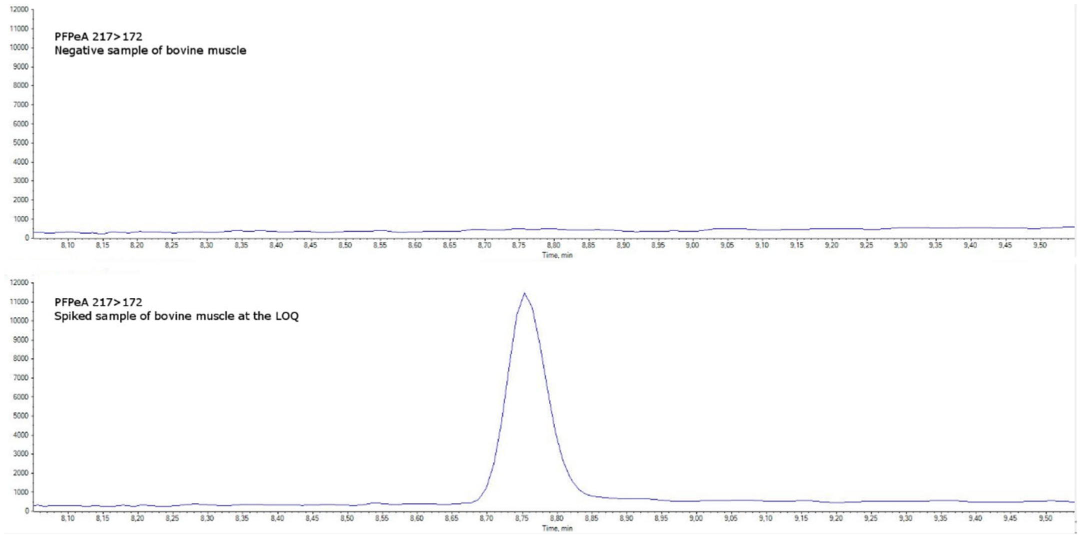

Figure S2. (PFPeA quantitative transition 217>172) Comparison between a negative sample of bovine muscle (upper chromatogram) and spiked sample of bovine muscle at the Limit of Quantification (lower chromatogram).

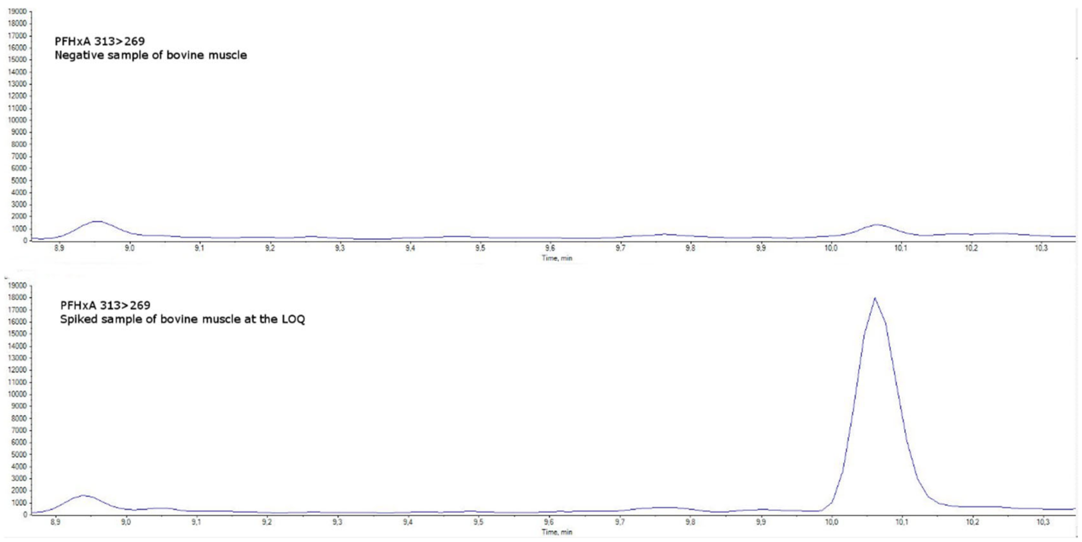

Figure S3. (PFHxA quantitative transition 313>269) Comparison between a negative sample of bovine muscle (upper chromatogram) and spiked sample of bovine muscle at the Limit of Quantification (lower chromatogram).

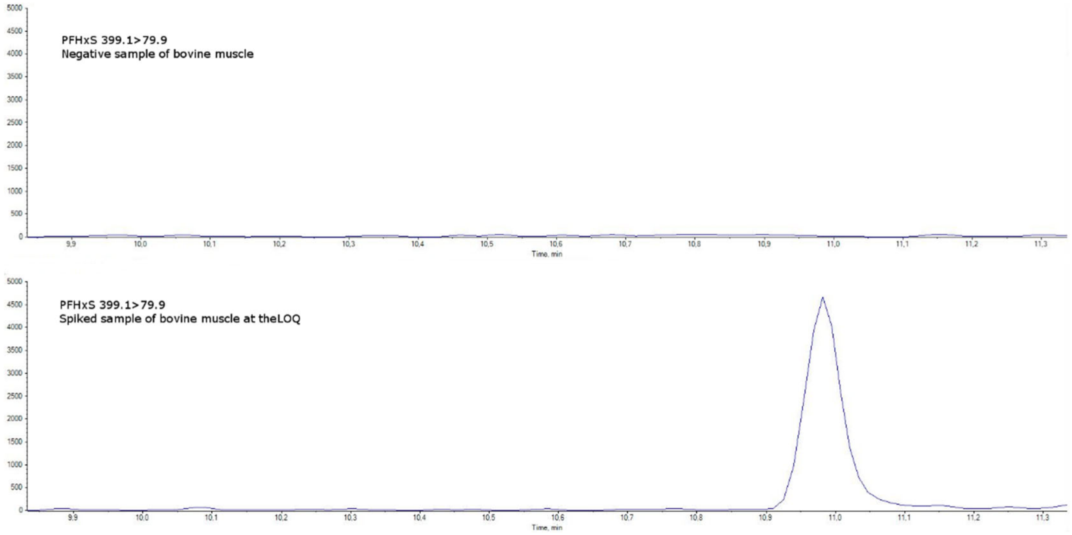

Figure S4. (PFHxS quantitative transition 399.1>79.9) Comparison between a negative sample of bovine muscle (upper chromatogram) and spiked sample of bovine muscle at the Limit of Quantification (lower chromatogram).

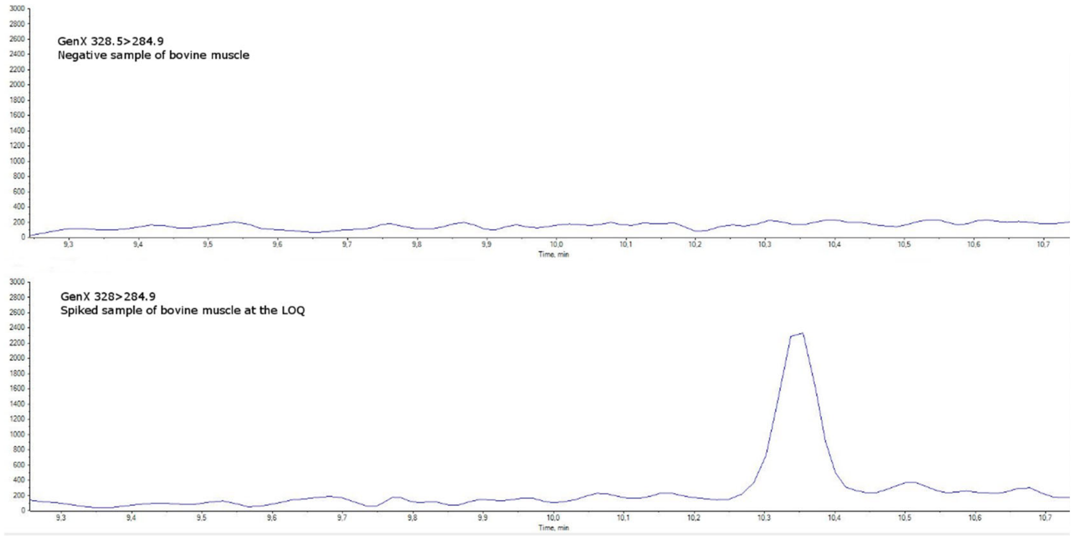

Figure S5. (GenX quantitative transition 328>284.9) Comparison between a negative sample of bovine muscle (upper chromatogram) and spiked sample of bovine muscle at the Limit of Quantification (lower chromatogram).

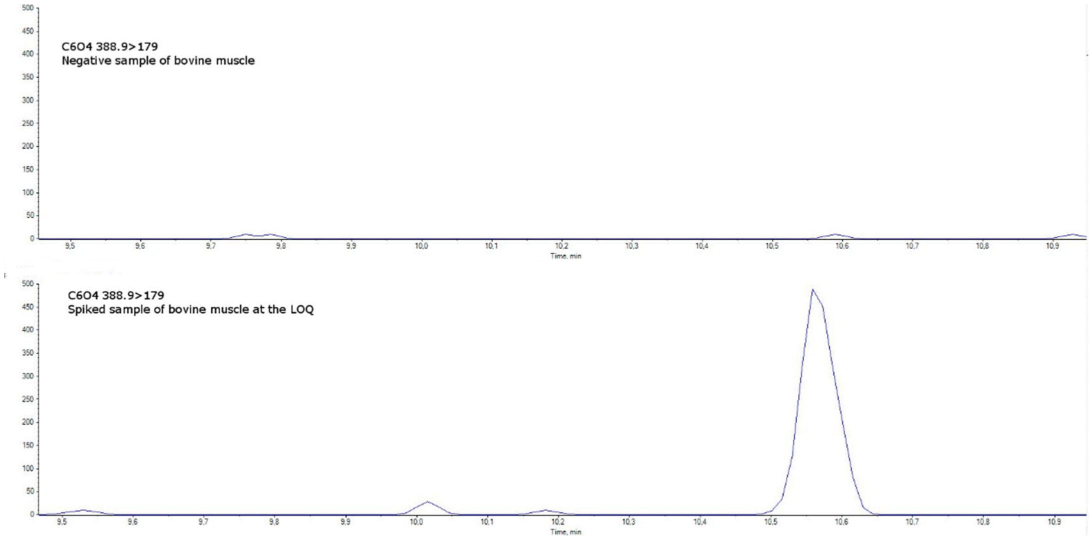

Figure S6. (C6O4 quantitative transition 388.9>179) Comparison between a negative sample of bovine muscle (upper chromatogram) and spiked sample of bovine muscle at the Limit of Quantification (lower chromatogram).

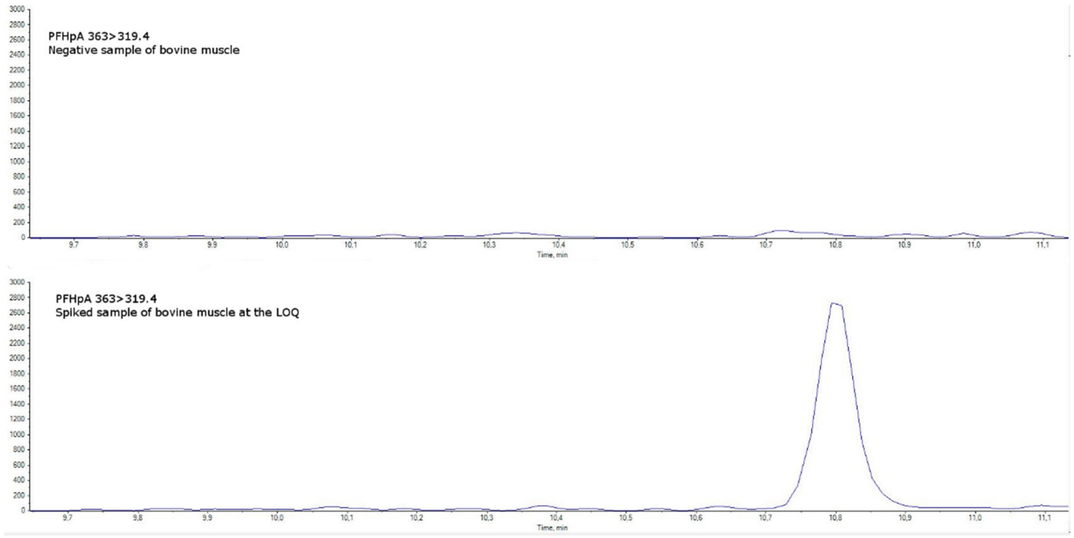

Figure S7. (PFHpA quantitative 363>319.4) Comparison between a negative sample of bovine muscle (upper chromatogram) and spiked sample of bovine muscle at the Limit of Quantification (lower chromatogram).

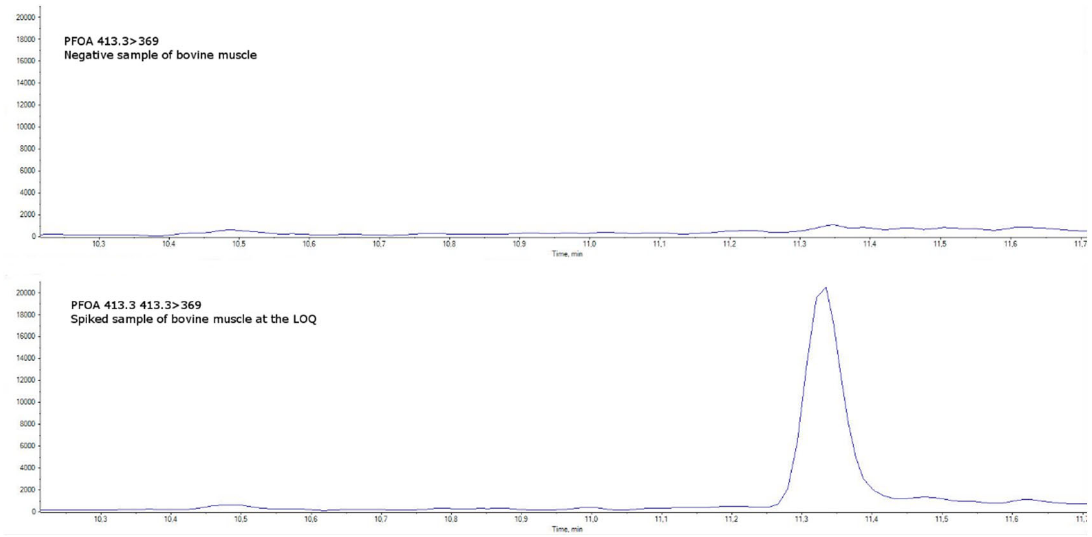

Figure S8. (PFOA quantitative 413.3>369) Comparison between a negative sample of bovine muscle (upper chromatogram) and spiked sample of bovine muscle at the Limit of Quantification (lower chromatogram).

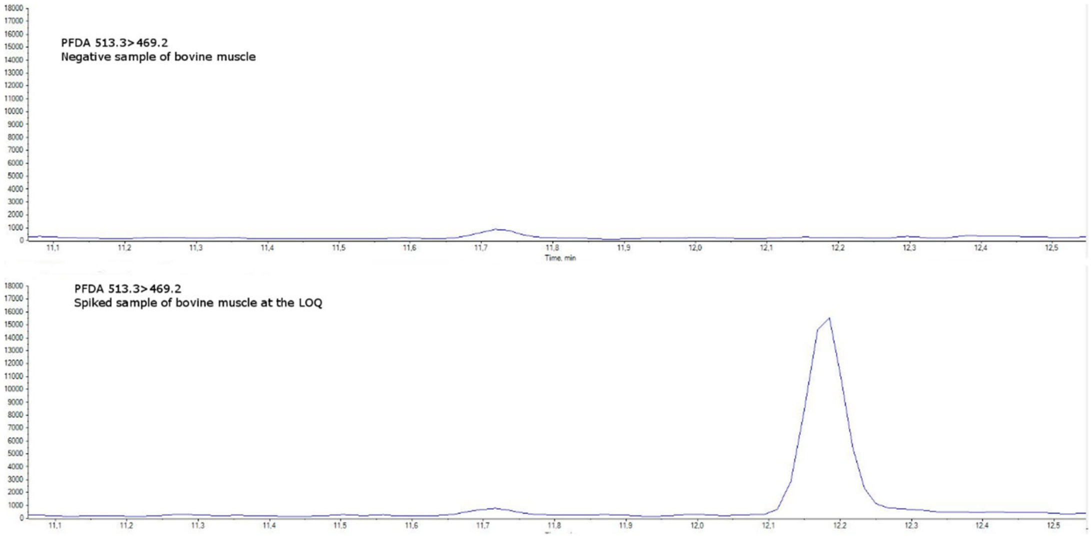

Figure S9. (PFDA quantitative 513.3>469.2) Comparison between a negative sample of bovine muscle (upper chromatogram) and spiked sample of bovine muscle at the Limit of Quantification (lower chromatogram).

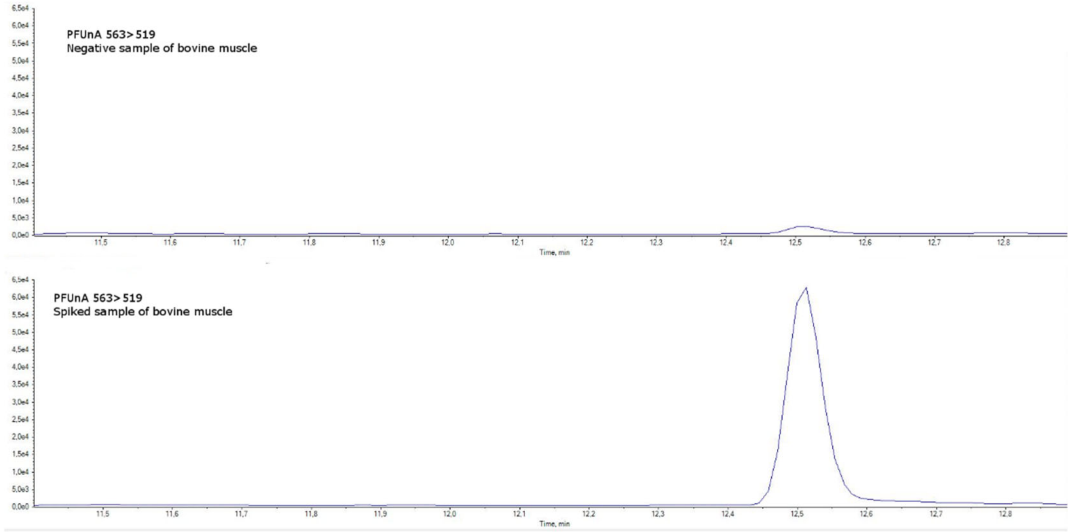

Figure S10. (PFUnA quantitative transition 563>519) Comparison between a negative sample of bovine muscle (upper chromatogram) and spiked sample of bovine muscle at the Limit of Quantification (lower chromatogram).

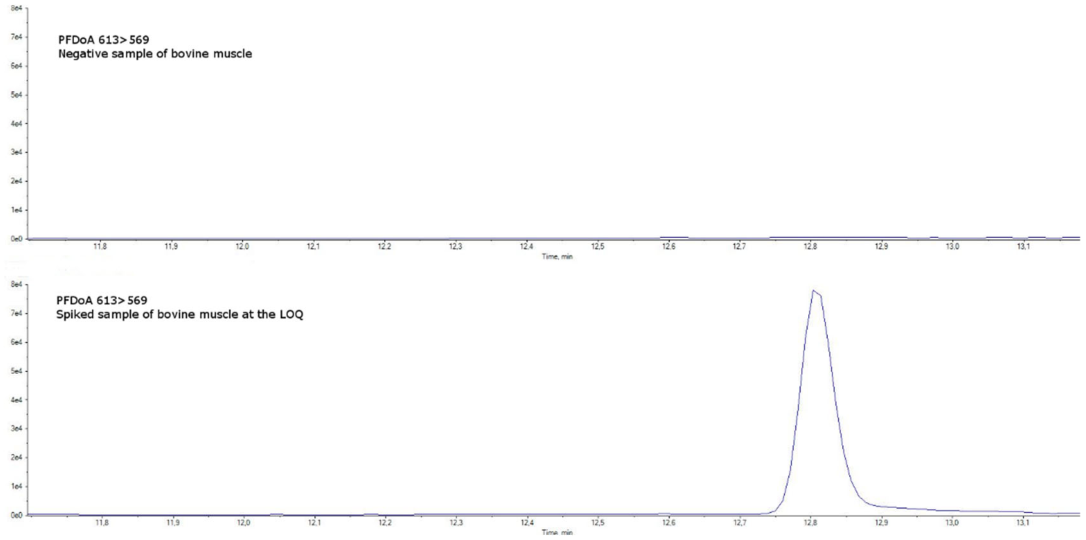

Figure S11. (PFDoA quantitative transition 613>569) Comparison between a negative sample of bovine muscle (upper chromatogram) and spiked sample of bovine muscle at the Limit of Quantification (lower chromatogram).
